# Psychometric validation of the Spanish HLS-EU-Q16 in Ecuador: evidence for health literacy assessment and public health education

**DOI:** 10.3389/fpubh.2026.1717946

**Published:** 2026-02-11

**Authors:** Gabriel Ortiz, Judith Francisco-Pérez, Víctor López-Guerra, Diana Maricela Vuele-Duma, Denny Caridad Ayora-Apolo, Angélica Rojas

**Affiliations:** 1 Departamento de Psicología, Universidad Técnica Particular de Loja, Loja, Ecuador; 2 Facultad de Salud y Bienestar, Grupo de Investigación en Salud Digital, Pontificia Universidad Católica del Ecuador, Quito, Ecuador; 3 Facultad de la Salud Humana, Universidad Nacional de Loja, Loja, Ecuador; 4 Programa de Psicología, Universidad Centroccidental Lisandro Alvarado, Barquisimeto, Venezuela

**Keywords:** disease prevention, Ecuador, education, health literacy, health promotion, HLS-EU-Q16, measurement invariance, psychometric validation

## Abstract

**Introduction:**

Health literacy (HL) is a key determinant of individual and public health outcomes, as it influences people's ability to access, understand, and apply health information for informed decision-making. Although the European Health Literacy Survey Questionnaire (HLS-EU-Q16) has been validated in several countries, no psychometric validation had previously been conducted in Ecuador.

**Methods:**

This study evaluated the psychometric properties of the Spanish version of the HLS-EU-Q16 in a sample of 612 Ecuadorian adults from the three main regions of the country. Confirmatory factor analysis (CFA) was conducted to examine the factorial structure. Internal consistency was assessed using Cronbach's alpha and McDonald's omega, measurement invariance was tested across sex, age, and area of residence, and known-groups validity was evaluated through group comparisons.

**Results:**

The CFA supported a three-factor model consistent with the theoretical framework of healthcare, disease prevention, and health promotion (χ^2^/df = 2.37, CFI = 0.990, TLI = 0.988, RMSEA = 0.039, SRMR = 0.049), with all factor loadings exceeding 0.50 and excellent model fit. Strict measurement invariance was confirmed across sex, age, and area of residence. The scale demonstrated excellent internal consistency (α = 0.92; ω = 0.94) and strong reliability across dimensions. Known-groups validity was supported, with higher HL levels observed among participants with postgraduate education, urban residence, and absence of financial hardship or chronic illness.

**Discussion:**

These findings confirm that the Spanish HLS-EU-Q16 is a valid, reliable, and invariant instrument for assessing health literacy in Ecuadorian adults. The availability of this tool provides a solid foundation for evidence-based health education, targeted interventions, and public health policies aimed at promoting equity and strengthening health literacy in Ecuador and Latin America.

## 1 Introduction

Health literacy (HL) is a key determinant of health promotion and disease prevention (1), as it shapes how people access, comprehend, and apply health information to make informed decisions (2–5). Higher levels of HL have been associated with more effective communication with healthcare professionals, better adherence to medical recommendations, and improved self-management of health (6).

HL is influenced not only by individual communication skills but also by the demands and complexity of society and healthcare systems (7). Empirical evidence links HL positively with medication adherence (8) and dietary compliance among individuals with type 2 diabetes (9). Furthermore, higher HL levels have been related to improved quality of life in patients with chronic diseases (9, 10), greater use of preventive services, and reduced healthcare costs (4).

Recent findings confirm the significant impact of HL on health outcomes (2). However, they also emphasize that barriers to adequate HL extend beyond access to reliable information. Sociocultural, educational, and economic factors strongly influence individuals' ability to understand and use health information effectively (11, 12). Thus, comprehensive research exploring HL across diverse age groups, educational levels, and sociocultural contexts is warranted (6). Addressing these differences is crucial for designing equitable and effective strategies that meet the real needs of populations.

In the case of Ecuador, the absence of validated instruments for assessing HL has limited the accurate estimation of population literacy levels, the identification of vulnerable or at-risk groups, and the evaluation of public health interventions aimed at improving HL. This gap has hindered evidence-based decision-making and the development of effective strategies to promote health equity in the country.

Recent meta-analytic evidence suggests that low HL is frequent in Latin America and the Caribbean; however, these estimates derive from heterogeneous studies using diverse methodologies and populations, including screening instruments, which limits direct comparability (13). For instance, a study in Brazil found that medication use, reliance on the Unified Health System (SUS), and educational attainment were significant predictors of HL (14). These findings suggest that HL determinants in the region are closely intertwined with healthcare system characteristics and socioeconomic conditions.

HL research is gradually shaping health policies in Latin America (15). Nevertheless, results vary substantially due to socioeconomic inequalities, cultural and linguistic barriers, and limited access to resources (16). Higher levels of education and employment have consistently been associated with better HL scores (17), reinforcing the need for HL research that explicitly addresses these disparities (18).

To ensure valid assessment of HL in different contexts, psychometrically robust and culturally sensitive instruments are required. The European Health Literacy Survey Questionnaire (HLS-EU-Q16) has demonstrated validity and reliability in several European countries and has been adapted in Latin America. However, its psychometric performance may vary depending on cultural, linguistic, and educational factors, highlighting the importance of validating the tool in each specific setting before implementation.

The HLS-EU-Q16 is particularly valued for its brevity and its focus on three core HL dimensions: healthcare, disease prevention, and health promotion (19, 20). Nevertheless, its factor structure has shown variability across countries, which has motivated extensive psychometric evaluations in both European and Latin American populations (21, 22).

In several contexts, factor analyses have confirmed the original three-factor model of healthcare, disease prevention, and health promotion (1). For example, in India, Hindi and Kannada versions demonstrated excellent internal consistency (α = 0.98 and α = 0.97), respectively (23). Similarly, in Bangladesh, both the Bengali HLS-EU-Q16 and HLS-EU-Q6 versions showed clear factor structures and high reliability (α = 0.934 and α = 0.857), respectively (24). In Romania, exploratory factor analysis on a representative sample of 1,622 individuals supported a three-factor model with good internal consistency (α = 0.84), while HL levels were influenced by age, sex, education, and self-perceived health (25).

Conversely, two-factor solutions have been reported. In Sweden, principal component analysis of the full HLS-EU-Q16 supported a two-factor structure with good internal consistency (α = 0.89) and temporal stability (Cohen's κ = 0.822) (26). In Mexico, García-Vera validated a shortened 12-item version (HLS-EU-Q12M) among hypertensive patients, identifying two main factors and reporting adequate reliability (α = 0.83) and test-retest stability (ICC = 0.94) (27).

Some studies have even supported a unidimensional model. In Brazil, the unidimensional structure was validated in adults, confirming the scale's consistency, accuracy, and stability (28). Likewise, in Sweden, a study focusing on multicultural contexts and parental HL identified a single factor explaining 37.3% of variance (21). In Spain, Nolasco et al. also reported a unidimensional model, where a single factor explained 79.1% of variance, supported by unidimensionality indices (UniCo = 0.998; ECV = 0.949; MIREAL = 0.191). Internal consistency was extremely high, with both McDonald's omega and Cronbach's alpha reaching 0.982 (29).

A four-factor solution was proposed in Iceland, encompassing medical, family, media, and lifestyle-related information processing as well as health information-seeking behavior, further supporting the instrument's adaptability (12).

This variability in factor structures, including one-, two-, three-, and four-factor models, highlights the sensitivity of the HLS-EU-Q16 to sociocultural and contextual differences. It reinforces the need for local validation before use. Despite strong international evidence for its robustness, Ecuador still lacks psychometric validation studies of the HLS-EU-Q16 in adults, limiting the identification of HL gaps and the development of tailored interventions to promote health equity.

Over the last decade, Ecuador has implemented health reforms emphasizing equity, inclusion, and the social determinants of health (30). These reforms led to the creation of the National Health System and the Integrated Public Health Network, which have strengthened primary care and helped reduce access inequalities (31, 32). Nevertheless, persistent disparities remain, particularly in curative, reproductive, and maternal services among Indigenous and Afro-Ecuadorian women (32, 33). Inequalities also persist across income, education, ethnicity, and geographic location (33, 34). Moreover, cultural and linguistic diversity continues to face challenges, while rural areas struggle with limited human resources, infrastructure shortages, and school closures that exacerbate territorial inequalities (35–39).

At the same time, measurement invariance has been scarcely examined in studies assessing health literacy instruments (40, 41). Addressing this gap is crucial, as invariance testing helps minimize biases derived from differential item functioning across groups (e.g., men and women), as well as between younger and older adults, and between rural and urban populations. Including this property in validation studies ensures that HL measures provide equitable and comparable results across diverse sociodemographic groups (41).

Considering these gaps, the present study aimed to evaluate the psychometric properties of the Spanish version of the HLS-EU-Q16 in Ecuadorian adults. First, the factorial structure of the scale was examined to confirm its dimensionality. Second, factorial invariance was tested across key sociodemographic variables (sex, age, education, and area of residence) to ensure comparability across groups. Third, the internal consistency of the instrument was analyzed to determine its reliability. Finally, construct validity was evaluated through group-contrast methods. The findings are expected to provide robust empirical evidence supporting the applicability of the HLS-EU-Q16 in Ecuador, inform the design of context-specific interventions, and guide public health policies aimed at strengthening health literacy and reducing inequalities in healthcare access and utilization.

## 2 Materials and methods

### 2.1 Type of study and research design

A non-experimental, cross-sectional, instrumental-psychometric study was conducted with the objective of analyzing the psychometric properties of a health literacy assessment instrument, following the methodological recommendations of Montero and León (42).

### 2.2 Participants

The study included a total of 612 participants, recruited through non-probabilistic convenience sampling, as used in similar validation studies in Latin America (13, 27, 28). Although efforts were made to include participants of different sexes, socioeconomic levels, ethnic backgrounds, geographic regions, and areas of residence (urban and rural), the sample was not representative of the general Ecuadorian adult population. This limitation should be considered when interpreting the findings, as the composition of the sample may introduce selection bias.

Participants residing in Ecuador were recruited through a multi-channel strategy designed to enhance geographic and demographic diversity. Recruitment was primarily conducted through digital outreach via WhatsApp, using groups and known contacts of the researchers and the Nursing Informatics Network Ecuador Chapter, who distributed the survey link. Additional recruitment occurred through in-person outreach in community settings, where trained assistants invited individuals outside pharmacies, hospitals, plazas, and shopping centers to participate. A snowball approach was also used, encouraging participants to share the invitation with other eligible adults. Throughout data collection, the regional origin of responses (Sierra, Costa, and Amazon) was tracked to guide additional outreach in underrepresented areas. Data were collected between February and April 2024, with weekly monitoring of demographic characteristics to adjust the strategy and improve representativeness.

The sample was predominantly composed of women (64.22%) and single individuals (63.40%). The mean age was 30.97 years (SD = 11.64; range = 18–81). Regarding education, 34.31% of participants reported having completed primary or secondary education, 21.41% technical education, 33.99% university studies, and 10.29% postgraduate studies. In terms of ethnic self-identification, most participants identified as Mestizo (86.76%), followed by Indigenous (5.72%), Afro-Ecuadorian (3.43%), Montubio (2.29%), and White (1.80%). Spanish was the native language for 98.04% of the participants.

Geographically, the majority resided in the highlands region (55.72%), followed by the Amazon (36.93%) and the coast (7.35%). Most participants lived in urban areas (77.12%) and cohabited with family members (89.05%). Regarding health status, most participants reported not suffering from chronic diseases (86.93%), although 60.46% indicated that at least one family member had a chronic condition. In relation to healthcare services, 47.71% of participants reported receiving care at facilities of the Ministry of Public Health, while 25.98% received care at the Ecuadorian Social Security Institute. Finally, 30.23% of the sample reported experiencing financial difficulties.

Further details of the sociodemographic characteristics of the sample are presented in Table 1.

**Table 1:** Socio-demographic characteristics of the study sample.

**Variable**		***N***	***%***
Gender	Female	393	64.22
	Male	219	35.78
Marital status	Single	388	63.40
	Married	178	29.08
	Divorced	39	6.37
	Widowed	7	1.14
Educational level	Primary/secondary	210	34.31
	Technical	131	21.41
	University/higher education	208	33.99
	Postgraduate	63	10.29
Ethnicity	Mixed	531	86.76
	Indigenous	35	5.72
	Afro-Ecuadorian	21	3.43
	Montubia	14	2.29
	White	11	1.80
Native language	Spanish	600	98.04
	Other	12	1.96
Region	Highlands	341	55.72
	Amazon	226	36.93
	Coast	45	7.35
Area	Urban	472	77.12
	Rural	140	22.88
Living conditions	With family	545	89.05
	Alone	56	9.15
	With friends	11	1.80
Chronic illness	No	532	86.93
	Yes	80	13.07
Relative with chronic illness	No	242	39.54
	Yes	370	60.46
Healthcare facility	Ministry of public health	292	47.71
	Social Security Institute	159	25.98
	Private clinic	122	19.93
	Other	39	6.37
Financial hardship	No	427	69.77
	Yes	185	30.23
Age	M (SD)	Min	Max
	30.97 (11.64)	18	81

n, sample size; %, percentage; M, mean; SD, standard deviation; Min, minimum; Max, maximum.

### 2.3 Instrument

#### 2.3.1 Sociodemographic data

A questionnaire was designed to collect basic sociodemographic information, including age, sex, marital status, educational level, ethnic self-identification, language, region of residence, area of residence (urban/rural), type of healthcare facility attended, presence of chronic disease, caregiver status of relatives with chronic conditions, and financial hardship. These variables were included to provide contextual information on the sample and to examine their potential influence on health literacy outcomes.

#### 2.3.2 European health literacy survey questionnaire (HLS-EU-Q16)

Health literacy was assessed using the HLS-EU-Q16, originally developed by Pelikan et al. (20) and validated in Spanish by Nolasco et al. (29). This self-administered questionnaire consists of 16 items that measure the perceived difficulty in accessing, understanding, evaluating, and applying health-related information across three domains: healthcare, disease prevention, and health promotion. Example items include:

“*How easy is it for you to find out where to get professional help when you are ill (e.g., doctor, pharmacist, psychologist)?”* and “*How easy is it for you to understand what your doctor says to you?”*

Responses were recorded on a five-point Likert-type scale ranging from 1 (*very difficult*) to 5 (*very easy*), with higher scores indicating greater health literacy.

### 2.4 Procedure

Participants were invited to complete the questionnaire on a voluntary and anonymous basis. Data collection was conducted between February and April 2024 through electronic forms administered via Google Forms, complemented by in-person recruitment in community settings (e.g., outside pharmacies, hospitals, and local markets) to enhance geographic and demographic diversity. Informed consent was obtained from all participants prior to participation, and the study was carried out in accordance with the ethical principles of the Declaration of Helsinki.

Before data collection, the Spanish version of the instrument was reviewed by three Ecuadorian experts (one psychologist with experience in psychometrics and two nurses, one of whom specialized in public health) to ensure linguistic and cultural adequacy for the Ecuadorian context. Minor wording adjustments were made to enhance clarity and cultural relevance. Subsequently, the instrument was pretested with 30 individuals who shared the sociodemographic characteristics of the main study sample to verify comprehension and cultural appropriateness of the items.

It should be noted that participation required proficiency in Spanish; therefore, individuals whose primary language was an Indigenous language may have been excluded, which could limit the generalizability of the findings to all cultural groups in Ecuador.

The research protocol was reviewed and approved by the Comité de Ética de la Investigación en Seres Humanos de la Pontificia Universidad Católica del Ecuador (Oficio CEISH-860-2023), in its session of October 26, 2023. The approval was granted for a period of 12 months, with the requirement to submit a partial report at 6 months and a final report upon completion of the study.

### 2.5 Data analysis

Statistical analyses were conducted in the R environment (version 4.4.1) using RStudio. First, descriptive statistics (means, standard deviations, skewness, and kurtosis) were calculated for the overall health literacy score, each item of the scale, and the three theoretical dimensions proposed by Sørensen et al. (1): healthcare, disease prevention, and health promotion.

Subsequently, a Confirmatory Factor Analysis (CFA) was performed to test five competing models corresponding to factorial structures reported in previous studies across different countries. Given the ordinal nature of the Likert-type items, all CFAs were estimated using the Weighted Least Squares Mean and Variance adjusted (WLSMV) method, which is recommended for categorical or ordinal data (43, 44). Model adequacy was assessed using robust fit indices, including the normed chi-square (χ^2^/df), Comparative Fit Index (CFI), Tucker–Lewis Index (TLI), Standardized Root Mean Square Residual (SRMR), and Root Mean Square Error of Approximation (RMSEA). The cut-off criteria were: χ^2^/df ≤ 3 for acceptable fit and ≤ 2 for optimal fit; CFI and TLI ≥ 0.90 for acceptable fit and ≥ 0.95 for optimal fit; RMSEA and SRMR ≤ 0.08 for acceptable fit and ≤ 0.05 for optimal fit (45, 46).

Internal consistency was examined using Cronbach's alpha (α) and McDonald's omega (ω) coefficients for both the total scale and the three dimensions. Reliability values above 0.70 were considered acceptable, and values above 0.80 indicated good internal consistency (47). This dual approach provides complementary information, with alpha estimating the lower bound of reliability and omega offering a more accurate estimate when item loadings differ across the scale.

Measurement invariance across gender, age groups (based on the median split), and area of residence (urban vs. rural) was examined using Multi-Group Confirmatory Factor Analysis (MG-CFA) with the WLSMV estimator. Four hierarchical models were tested sequentially: configural invariance (same factor structure across groups), metric invariance (equal factor loadings), scalar invariance (equal loadings and intercepts), and strict invariance (equal loadings, intercepts, and error variances). Model comparisons were evaluated using changes in fit indices (ΔCFI and ΔRMSEA), with thresholds of ΔCFI ≤ 0.010 and ΔRMSEA ≤ 0.015 indicating acceptable invariance (48).

Construct validity was assessed using the known-groups method, comparing health literacy levels and the three domains across categorical sociodemographic variables (sex, age group, socioeconomic level, region, and urban/rural residence) through ANOVA and Student's *t*-tests. When significant differences were detected, Tukey's HSD *post hoc* test was applied to identify specific group differences. Effect sizes were interpreted following Cohen's (49) criteria, where values of ^*^d^*^ ≈ 0.20, 0.50, and 0.80 indicate small, medium, and large effects, respectively, and partial η^2^ values of 0.01, 0.06, and 0.14 correspond to small, medium, and large effects.

Finally, a linear regression analysis was conducted to identify predictors of health literacy. Predictor variables included those that showed statistically significant differences in the ANOVA or *t*-tests.

## 3 Results

### 3.1 Descriptive analysis

The descriptive analysis of the HLS-EU-Q16 indicated that participants reported a moderate level of health literacy, with a mean item score of 3.42 (SD = 0.54).

When examining the three theoretical dimensions, Health Promotion obtained the highest mean score (M = 3.54, SD = 0.63), indicating greater confidence in the ability to maintain and improve health through information use. Healthcare followed with an intermediate mean score (M = 3.41, SD = 0.59), whereas Disease Prevention showed the lowest mean (M = 3.35, SD = 0.59), highlighting potential limitations in recognizing health risks and engaging in preventive actions. These findings underscore the need for interventions specifically designed to strengthen preventive health literacy (see Table 2).

**Table 2:** Descriptive statistics of the HLS-EU-Q16 and its three theoretical dimensions.

**Variable**	**M**	**SD**	**Skewness**	**Kurtosis**	**Min**	**Max**
HL 1	3.25	0.87	−0.05	0.37	1.00	5.00
HL 2	3.22	0.86	−0.14	0.16	1.00	5.00
HL 3	3.49	0.77	−0.27	0.49	1.00	5.00
HL 4	3.62	0.74	−0.30	0.36	1.00	5.00
HL 5	3.29	0.79	−0.19	0.43	1.00	5.00
HL 6	3.38	0.76	−0.30	0.82	1.00	5.00
HL 7	3.63	0.74	−0.46	0.37	1.00	5.00
HL 8	3.10	0.89	−0.24	0.23	1.00	5.00
HL 9	3.64	0.76	−0.44	0.63	1.00	5.00
HL 10	3.50	0.78	−0.45	0.46	1.00	5.00
HL 11	3.16	0.80	−0.09	0.32	1.00	5.00
HL 12	3.34	0.76	−0.23	0.32	1.00	5.00
HL 13	3.56	0.77	−0.51	0.61	1.00	5.00
HL 14	3.53	0.73	−0.09	0.14	1.00	5.00
HL 15	3.47	0.72	−0.08	0.15	1.00	5.00
HL 16	3.60	0.80	−0.49	0.59	1.00	5.00
Healthcare	3.41	0.59	0.16	0.37	1.86	5.00
Disease prevention	3.35	0.59	0.06	0.19	1.40	5.00
Health promotion	3.54	0.63	−0.03	0.35	1.00	5.00
Health literacy	3.42	0.54	0.25	0.16	1.88	5.00

M, mean; SD, standard deviation; Sk, skewness; Ku, kurtosis; Min, minimum; Max, maximum. HL1–HL16 correspond to the 16 items of the European Health Literacy Survey Questionnaire (HLS-EU-Q16), assessing perceived ease or difficulty in finding, understanding, evaluating, and applying health information. A complete list of item statements is provided in Supplementary Table S1.

At the item level, meaningful variations were observed. The highest score corresponded to item 9 (M = 3.64), which evaluates the ability to understand health warnings about risky habits such as smoking, physical inactivity, or excessive alcohol consumption. This pattern suggests that participants are particularly receptive to concrete and frequently disseminated health messages, likely due to the emphasis placed on such warnings in public health campaigns. By contrast, the lowest score was found in item 8 (M = 3.10), related to finding information on mental health problems such as stress or depression. This result points to persistent barriers in accessing reliable information on mental health, which may reflect stigma, limited dissemination, or insufficient availability of specialized services.

Finally, the distributional properties of the items showed skewness values between −0.51 and −0.05 and kurtosis values between 0.14 and 0.82, indicating that the responses were approximately normally distributed and covered the full range of the scale (1.00–5.00). These characteristics support the adequacy of the data for subsequent multivariate analyses.

### 3.2 Confirmatory factor analysis

To evaluate the factorial validity of the HLS-EU-Q16, a series of CFAs were conducted using the Weighted Least Squares Mean and Variance adjusted (WLSMV) estimation method, which is recommended for ordinal data derived from Likert-type items. Five competing models were tested, based on both empirical findings and theoretical frameworks (see Table 3).

**Table 3:** Goodness-of-fit indices for alternative confirmatory factor analysis models.

**Model**	**CMIN/DF**	**CFI**	**TLI**	**RMSEA**	**SRMR**
M1	3.618	0.978	0.975	0.057 [0.051, 0.063]	0.067
M2	2.545	0.988	0.986	0.042 [0.036, 0.048]	0.052
M3	**2.366**	**0.990**	**0.988**	**0.039 [0.033, 0.046]**	**0.049**
M4	2.942	0.987	0.984	0.045 [0.039, 0.051]	0.052
M5	3.145	0.986	0.981	0.048 [0.040, 0.057]	0.053

CMIN/D, ratio square (χ^2^) by degrees of freedom; CFI, Bentler comparative fit index; TLI, Tucker–Lewis index; SRMR, standardized root mean squared residual; RMSEA, Root mean square error of approximation.

Bold values indicate acceptable or good model fit according to commonly recommended cutoff criteria.

Model 1: One-Factor Model. Proposed by Nolasco et al. (29) and Mekhail et al. (21), this model assumes that all items load onto a single latent Health Literacy factor.

Model 2: Two-Factor Model. Supported by Francisco-Pérez et al. (41) in their exploratory factor analysis, this model distinguishes between *Healthcare* (items 1–7) and *Disease Prevention/Health Promotion* (items 8–16).

Model 3: Three-Factor Model. Consistent with the conceptual framework proposed by Sørensen et al. (1) and also reported by Francisco-Pérez et al. (41), this model specifies three correlated factors: *Healthcare* (items 1–7), *Disease Prevention* (items 8–12), and *Health Promotion* (items 13–16).

Model 4: Four-Factor Model. Reported by Gustafsdottir et al. (12), this solution includes: (a) Processing and Using Information from Doctors (items 3, 5, 6, 7); (b) Processing and Using Information from Family and Media (items 11, 12, 14, 15); (c) Processing Information Related to Healthy Lifestyle (items 4, 9, 10, 13, 16); and (d) Finding Information about Health Problems/Illnesses (items 1, 2, 8).

Model 5: Four-Dimension Information-Processing Model. Derived from Sørensen (1) and operationalized by Falcón Romero et al. (50), this model organizes items into four sequential dimensions: *Access* (items 1, 5, 9), *Understand* (items 2, 6, 10), *Process* (items 3, 7, 11), and *Apply* (items 4, 8, 12).

As presented in Table 3, all estimated models exhibited acceptable levels of fit. However, the three-factor model (Model 3) provided the most favorable indices: χ^2^/df = 2.366, CFI = 0.990, TLI = 0.988, RMSEA = 0.039 (90% CI: 0.033–0.046), and SRMR = 0.049. These values meet the recommended cut-off criteria proposed by Hu and Bentler (45), supporting the adequacy of this factorial solution.

**Figure 1:**
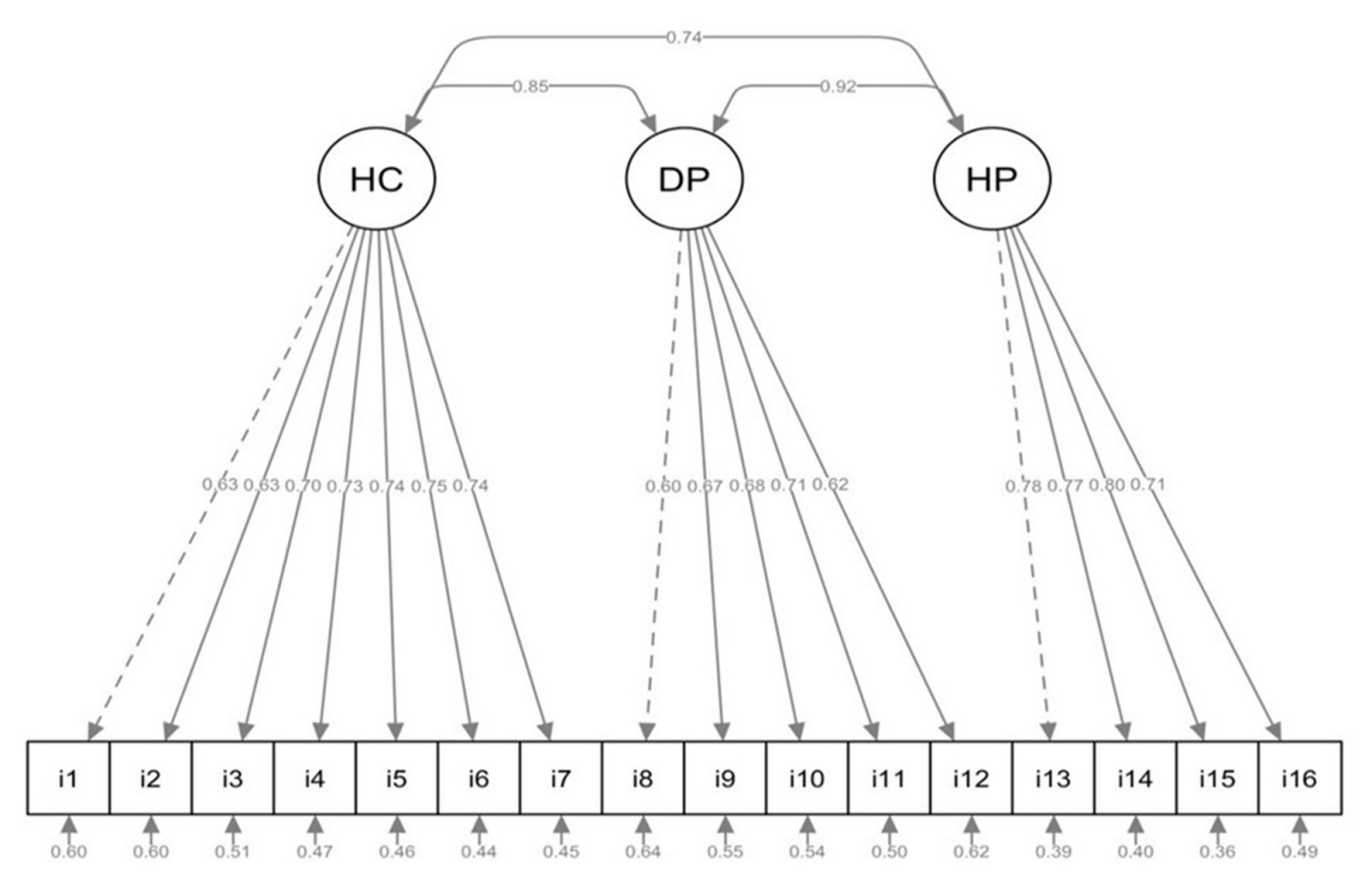
Confirmatory factor analysis Model 3 of the HLS-EU-Q16. The diagram illustrates the three dimensions (Healthcare, Disease Prevention, and Health Promotion) with their respective standardized loadings, correlations among factors, and error variances. This model showed the best fit to the data.

Additionally, all standardized factor loadings were positive and exceeded the recommended threshold of 0.50 (see Figure 1). Within the *Healthcare* factor (items 1–7), all loadings were above.60, indicating strong associations. In the *Disease Prevention* dimension (items 8–12), the lowest loadings corresponded to items i8 (0.60) and i12 (0.62), while in the *Health Promotion* dimension (items 13–16), item i14 (0.70) was the lowest; however, all remained within acceptable limits.

The correlations among the three latent dimensions were high: Healthcare–Disease Prevention (*r* = 0.85, *p* < 0.001), Healthcare–Health Promotion (*r* = 0.74, *p* < 0.001), and Disease Prevention–Health Promotion (*r* = 0.92, *p* < 0.001). The particularly strong association between Disease Prevention and Health Promotion suggests substantial overlap between these domains, indicating that they may reflect closely related aspects of a broader underlying construct of health literacy. Although the three-factor model provided the best statistical fit, this high intercorrelation pattern should be interpreted with caution, as it points toward an essentially unidimensional latent structure.

Taken together, these findings provide strong empirical support for the theoretical three-dimensional structure of health literacy originally proposed by Sørensen et al. (1). However, the high correlations observed among the latent dimensions particularly between Disease Prevention and Health Promotion suggest that these domains may reflect closely related aspects of a broader underlying construct. Thus, while the three-factor model demonstrated the best statistical fit and theoretical consistency, the pattern of intercorrelations also points toward an essentially unidimensional latent structure. Overall, the scale demonstrates adequate psychometric properties to capture three interrelated but conceptually meaningful domains, reflecting the multidimensional nature of health literacy in public health contexts.

### 3.3 Factorial invariance

The factorial invariance of the HLS-EU-Q16 was assessed across sex, age groups ( ≤ 27 vs. ≥28 years), and area (urban vs. rural) using Multi-Group Confirmatory Factor Analysis (MG-CFA). Four increasingly restrictive models were tested sequentially: configural, metric, scalar, and strict invariance. For each level of invariance, model fit was evaluated using the Comparative Fit Index (CFI), the Root Mean Square Error of Approximation (RMSEA) with 90% confidence intervals, and the Standardized Root Mean Square Residual (SRMR). Model comparisons were evaluated using changes in these fit indices (ΔCFI, ΔRMSEA), interpreted according to the guidelines of Chen (48), with thresholds of ΔCFI ≤ 0.010 and ΔRMSEA ≤ 0.015 indicating acceptable invariance (see Table 4).

**Table 4:** Factorial invariance of the HLS-EU-Q16 across the total sample, sex, age groups, and area of residence.

**Model**	**χ2**	**Df**	**C-M**	**Δχ2**	**Δdf**	**CFI**	**ΔCFI**	**RMR**	**RMSEA [CI 90%]**	**ΔRMSEA**
Entire group	239.005	101	–	–	–	0.990	–	0.031	0.039 [0.033, 0.046]	–
Male	133.596	101	–	–	–	0.990	–	0.042	0.043 [0.019, 0.061]	–
Female	194.687	101	–	–	–	1.000	–	0.030	0.037 [0.029, 0.045]	–
MC	299.80	202	–	–	–	0.990	–	0.032	0.039 [0.029, 0.048]	–
MM	295.03	215	MM-MC	4.77	13	0.989	0.001	0.036	0.040 [0.028, 0.051]	0.001
SC	322.09	228	SC-MM	27.06	13	0.987	0.002	0.038	0.042 [0.031, 0.052]	0.002
ST	337.05	244	ST-SC	14.96	16	0.987	0.000	0.040	0.041 [0.029, 0.051]	0.001
≤ 27 years	182.266	101	–	–	–	0.990	–	0.030	0.038 [0.029, 0.047]	–
≥28 years	156.182	101	–	–	–	0.989	–	0.041	0.042 [0.029, 0.055]	–
MC	325.62	202	–	–	–	1.000	–	0.033	0.040 [0.032, 0.048]	–
MM	333.48	215	MM-MC	7.86	13	0.998	0.002	0.039	0.045 [0.035, 0.054]	0.005
SC	354.14	228	SC-MM	20.66	13	0.997	0.000	0.040	0.045 [0.036, 0.054]	0.000
ST	374.51	244	ST-SC	20.37	16	0.997	0.000	0.043	0.045 [0.036, 0.054]	0.000
Urban	197.118	101	–	–	–	0.990	–	0.034	0.040 [0.031, 0.048]	–
Rural	132.806	101	–	–	–	1.000	–	0.035	0.043 [0.019, 0.062]	–
MC	328.25	202	–	–	–	1.000	–	0.032	0.040 [0.032, 0.048]	–
MM	287.29	215	MM-MC	40.96	13	1.000	0.000	0.034	0.037 [0.024, 0.047]	0.004
SC	309.03	228	SC-MM	21.74	13	1.000	0.000	0.035	0.038 [0.026, 0.048]	0.001
ST	319.87	244	ST-SC	10.84	16	1.000	0.000	0.037	0.036 [0.024, 0.047]	0.001

χ^2^, chi-square; df, degrees of freedom; C-M, comparison of factorial invariance models; CFI, comparative fit index; SRMR, standardized root mean square residual; RMSEA, root mean square error of approximation; Δ, change; MC, configural model; MM, metric model; SC, scalar model; ST, strict model.

Model comparisons were conducted relative to the configural baseline model, and all ΔCFI and ΔRMSEA values were computed as absolute differences. Positive Δχ^2^ and minimal changes in fit indices confirmed the adequacy of the progressive constraints across models.

Across sex, results supported full measurement invariance. The configural model demonstrated an adequate fit (CFI = 0.990; RMSEA = 0.039). The metric model (ΔCFI = 0.001; ΔRMSEA = 0.001), scalar model (ΔCFI = 0.002; ΔRMSEA = 0.002), and strict model (ΔCFI = 0.000; ΔRMSEA = 0.001) all exhibited negligible differences relative to the baseline, confirming invariance at all levels.

For age groups, invariance was also established. The configural model showed satisfactory fit (CFI = 1.000; RMSEA = 0.040), while the metric (ΔCFI = 0.002; ΔRMSEA = 0.005), scalar (ΔCFI = 0.000; ΔRMSEA = 0.000), and strict models (ΔCFI = 0.000; ΔRMSEA = 0.000) remained within acceptable thresholds, indicating that the measurement structure was equivalent across younger and older participants.

Finally, analyses by area of residence (urban vs. rural) indicated strict factorial invariance. The configural model yielded CFI = 1.000 and RMSEA = 0.040, and subsequent metric (ΔCFI = 0.000; ΔRMSEA = 0.004), scalar (ΔCFI = 0.000; ΔRMSEA = 0.001), and strict models (ΔCFI = 0.000; ΔRMSEA = 0.001) confirmed minimal changes, supporting invariance across residential areas.

Taken together, all invariance comparisons met the conventional criteria (ΔCFI ≤ 0.010; ΔRMSEA ≤ 0.015), demonstrating that the factorial structure of the HLS-EU-Q16 is stable across sex, age, and area of residence. These findings provide robust evidence of strict measurement invariance, supporting the equivalence of the scale and confirming that it consistently assesses the three health literacy domains: Healthcare, Disease Prevention, and Health Promotion across diverse demographic groups.

### 3.4 Reliability

The reliability of the HLS-EU-Q16 and its three dimensions was evaluated using Cronbach's alpha and McDonald's omega coefficients. The full scale demonstrated excellent internal consistency (α = 0.92; ω = 0.94). At the dimensional level, reliability values were also strong**:** Healthcare (α = 0.87; ω = 0.91), Disease Prevention (α = 0.79; ω = 0.84), and Health Promotion (α = 0.85; ω = 0.87). All coefficients exceeded the commonly accepted threshold of 0.70, indicating that both the total scale and its subscales are reliable measures of health literacy in the Ecuadorian population.

### 3.5 Construct validity through contrasted groups

The contrasted groups analysis provided additional support for the construct validity of the HLS-EU-Q16. Significant differences were observed across sociodemographic and health-related variables (see Table 5).

**Table 5:** Comparison of health literacy and its dimensions by sociodemographic variables.

**Characteristics**	**Dimensions**															
	**Health literacy**				**Healthcare**				**Disease prevention**				**Health promotion**			
	**M (SD)**	***F*** **/** ***t***	***p*** **-Value**	***d*****/**η	**M (SD)**	***F*** **/** ***t***	***p*** **-Value**	***d*****/**η	**M (SD)**	***F*** **/** ***t***	***p*** **-Value**	***d*****/**η	**M (SD)**	***F*** **/** ***t***	***p*** **-Value**	***d*****/**η
**Gender**																
Female	3.40 (0.50)	−1.11	0.27	−0.11	3.38 (0.56)	−1.53	0.13	−0.13	3.34 (0.57)	−0.56	0.58	−0.05	3.53 (0.59)	−0.65	0.52	−0.05
Male	3.46 (0.59)				3.46 (0.65)				3.37 (0.62)				3.56 (0.69)			
**Marital status**																
Single	3.42 (0.52)	0.29	0.83	0.00	3.41 (0.57)	0.60	0.61	0.00	3.34 (0.56)	0.29	0.84	0.00	3.53 (0.62)	0.80	0.50	0.00
Married	3.43 (0.58)				3.40 (0.64)				3.36 (0.64)				3.59 (0.64)			
Divorced	3.43 (0.54)				3.49 (0.63)				3.35 (0.58)				3.44 (0.67)			
Widowed	3.60 (0.30)				3.63 (0.43)				3.54 (0.36)				3.61 (0.45)			
**Educational level**																
Primary/secondary	3.34 (0.53)	**9.91**	**0.00**	**0.05**	3.31 (0.57)	**12.27**	**0.00**	**0.06**	3.28 (0.60)	**5.34**	**0.00**	**0.03**	3.47 (0.65)	**5.49**	**0.00**	**0.03**
Technical	3.38 (0.51)				3.33 (0.55)				3.35 (0.54)				3.49 (0.63)			
University/higher education	3.44 (0.49)				3.44 (0.55)				3.34 (0.56)				3.55 (0.56)			
Postgraduate	3.74 (0.65)				3.80 (0.73)				3.61 (0.65)				3.82 (0.70)			
**Ethnicity**																
Mixed	3.43 (0.54)	0.36	0.84	0.00	3.42 (0.60)	0.23	0.92	0.00	3.36 (0.60)	0.90	0.46	0.01	3.55 (0.63)	0.88	0.48	0.01
Indigenous	3.34 (0.48)				3.35 (0.56)				3.21 (0.47)				3.49 (0.56)			
Afro-Ecuadorian	3.39 (0.53)				3.35 (0.60)				3.43 (0.49)				3.40 (0.69)			
Montubia	3.38 (0.55)				3.40 (0.50)				3.27 (0.64)				3.50 (0.61)			
White	3.34 (0.42)				3.49 (0.43)				3.18 (0.46)				3.27 (0.60)			
**Native language**																
Spanish	3.42 (0.54)	0.34	0.74	0.11	3.41 (0.59)	0.77	0.46	0.27	3.35 (0.59)	−0.36	0.72	−0.08	3.54 (0.63)	0.10	0.92	0.03
Other	3.36 (0.61)				3.25 (0.73)				3.40 (0.50)				3.52 (0.65)			
**Region**																
Highlands	3.49 (0.56)	**6.28**	**0.00**	**0.02**	3.49 (0.60)	**8.01**	**0.00**	**0.03**	3.40 (0.60)	**4.69**	**0.01**	**0.02**	3.58 (0.66)	2.65	0.07	0.01
Coast	3.32 (0.49)				3.30 (0.57)				3.25 (0.56)				3.46 (0.59)			
Amazon	3.44 (0.51)				3.36 (0.59)				3.42 (0.53)				3.62 (0.53)			
Urban	3.45 (0.55)	**2.07**	**0.04**	**0.19**	3.44 (0.61)	**2.53**	**0.01**	**0.22**	3.36 (0.61)	1.31	0.19	0.10	3.56 (0.65)	1.35	0.18	0.13
Rural	3.35 (0.47)				3.31 (0.53)				3.30 (0.52)				3.48 (0.55)			
**Living conditions**																
With family	3.41 (0.53)	1.98	0.14	0.01	3.39 (0.58)	2.53	0.08	0.01	3.34 (0.59)	0.40	0.67	0.00	3.52 (0.62)	2.50	0.08	0.01
Alone	3.53 (0.59)				3.53 (0.74)				3.41 (0.55)				3.68 (0.63)			
With friends	3.61 (0.54)				3.69 (0.61)				3.36 (0.48)				3.77 (0.65)			
**Chronic illness**																
No	3.44 (0.52)	**2.23**	**0.03**	**0.28**	3.43 (0.57)	1.69	0.09	0.24	3.37 (0.58)	1.93	0.06	0.26	**3.57 (0.61)**	**2.61**	**0.01**	**0.35**
Yes	3.29 (0.61)				3.29 (0.70)				3.22 (0.63)				**3.35 (0.70)**			
**Relative with chronic illness**																
No	3.42 (0.56)	−0.16	0.88	0.02	3.41 (0.61)	−0.19	0.85	0.02	3.35 (0.61)	−0.14	0.89	0.00	3.54 (0.66)	−0.06	0.96	0.00
Yes	3.43 (0.50)				3.42 (0.56)				3.35 (0.54)				3.54 (0.58)			
**Healthcare facility**																
Ministry of public health	3.36 (0.50)	**3.63**	**0.01**	**0.02**	3.30 (0.57)	**8.37**	**0.00**	**0.04**	3.30 (0.56)	1.47	0.22	0.01	3.52 (0.58)	0.21	0.89	0.00
Social Security Institute	3.44 (0.50)				3.43 (0.56)				3.38 (0.55)				3.54 (0.57)			
Private clinic	3.53 (0.62)				3.59 (0.64)				3.40 (0.67)				3.57 (0.72)			
Other	3.53 (0.62)				3.58 (0.62)				3.43 (0.67)				3.58 (0.90)			
**Financial hardship**																
No	3.51 (0.52)	**6.55**	**0.00**	**0.58**	3.51 (0.58)	**6.49**	**0.00**	**0.57**	3.43 (0.56)	**5.16**	**0.00**	**0.47**	3.63 (0.60)	**5.24**	**0.00**	**0.47**
Yes	3.21 (0.52)				3.18 (0.57)				3.16 (0.60)				3.34 (0.64)			

Results for the three HLS-EU-Q16 dimensions: Healthcare, Disease Prevention, and Health Promotion are presented to preserve the theoretical integrity of the multidimensional structure proposed by Sørensen et al. This approach allows a more comprehensive interpretation of health literacy patterns across demographic subgroups. All abbreviations are defined for clarity.

HL, health literacy; M, mean; SD, standard deviation; p, significance level; F/t, F statistic (ANOVA) or t statistic (Student's t-test), depending on the number of groups; d/η, effect size Cohen's d for two groups; η^2^ for multiple group.

Bold values indicate statistically significant group differences (*p* < 0.05), with corresponding effect sizes (Cohen's d or η^2^) reported.

For educational level, differences in the total health literacy score reached statistical significance with a small effect size (*F* = 9.91, *p* < 0.01, η^2^ = 0.05). *Post hoc* tests indicated that participants with postgraduate education reported higher levels of health literacy than those with lower educational attainment. This pattern was consistent across all three dimensions and was particularly pronounced in Healthcare, where the effect size was medium (*F* = 12.27, *p* < 0.01, η^2^ = 0.06).

Differences by region of residence were also significant, albeit with small effect sizes. Participants from the highlands reported higher scores on the total scale compared to those from the Amazon (*F* = 2.07, *p* < 0.05, η^2^ = 0.02). These contrasts were clearer in Healthcare (*F* = 8.01, *p* < 0.01, η^2^ = 0.03) and Disease Prevention (*F* = 4.69, *p* < 0.01, η^2^ = 0.02).

Regarding area of residence, urban participants scored higher than rural participants on both the total scale (*t* = 2.07, *p* < 0.05, *d* = 0.19) and the Healthcare dimension (*t* = 2.53, *p* < 0.01, *d* = 0.22), both representing small effects.

Participants reporting a chronic illness obtained lower health literacy scores (M = 3.29) than those without a chronic illness (M = 3.44), with a small effect size (*t* = 2.23, *p* < 0.05, *d* = 0.28). This contrast was more evident in Health Promotion, where the effect size approached medium magnitude (*t* = 2.61, *p* < 0.01, *d* = 0.35).

In terms of type of healthcare facility, significant group differences with a small effect size were identified (*F* = 3.63, *p* < 0.01, η^2^ = 0.02). *Post hoc* tests showed that users of private clinics (M = 3.53) and other facilities (M = 3.58) scored higher than those attending the Ministry of Public Health (M = 3.36, *p* < 0.05). The contrast was strongest in Healthcare, with a small effect size (*F* = 8.37, *p* < 0.01, η^2^ = 0.04).

Finally, participants experiencing financial hardship demonstrated markedly lower levels of health literacy. The difference was medium in the total scale (*t* = 6.55, *p* < 0.01, *d* = 0.58) and in Healthcare (*t* = 6.49, *p* < 0.01, *d* = 0.57). In Disease Prevention (*t* = 5.16, *p* < 0.01, *d* = 0.47) and Health Promotion (*t* = 5.24, *p* < 0.01, *d* = 0.47), effects were small to medium.

Overall, the scale demonstrated a consistent ability to discriminate across sociodemographic and health-related groups, providing robust evidence of construct validity through contrasted groups in the Ecuadorian adult population (see Table 5).

To complement these analyses, a linear regression was conducted to identify independent predictors of Health Literacy. The overall model was statistically significant [*F* (11, 600) = 8.19, *p* < 0.001] and explained 11.5% of the variance (*R*^2^ = 0.131, adjusted *R*^2^ = 0.115**)**. Postgraduate education was positively associated with higher Health Literacy compared to basic/secondary education (β = 0.18, *p* < 0.001, sr^2^ = 0.039), while having a chronic disease (β = −0.11, *p* < 0.01, sr^2^ = 0.012) and experiencing financial hardship (β = −0.25, *p* < 0.001, sr^2^ = 0.054) were significantly associated with lower levels. Financial hardship emerged as the strongest unique predictor, accounting for 5.4% of the variance. No significant associations were found for technical or university education, region, geographical area, or type of healthcare facility (all *p* > 0.05).

Although the explained variance was modest (adjusted *R*^2^ = 0.115), these findings highlight the importance of socioeconomic and health status in shaping Health Literacy. Additional determinants such as prior health experiences, social support, cognitive abilities, and media literacy should be addressed in future research to capture the multifactorial nature of Health Literacy.

## 4 Discussion

This study evaluated the psychometric properties of the Spanish version of the HLS-EU-Q16 in a sample of Ecuadorian adults, addressing the lack of validated instruments to assess health literacy (HL) in the country. The findings provide solid evidence of validity and reliability, supporting the three-factor structure proposed by Sørensen et al. (1) and confirming excellent internal consistency across the global scale and its dimensions. The confirmation of measurement invariance across sociodemographic groups further demonstrated the stability of the instrument, while differences by education, residence, and health conditions underscored the influence of social and health-related factors on HL. However, the very high correlations observed among the latent dimensions, particularly between Disease Prevention and Health Promotion (*r* = 0.92, *p* < 0.001), suggest that these subscales are strongly interrelated. Conceptually, Sørensen's framework differentiates these domains as distinct components of HL focusing, respectively on preventive and promotive behaviors, yet the empirical overlap observed indicates that they may reflect closely connected aspects of a broader construct. Thus, while the three-factor model was retained for theoretical and cross-cultural consistency, the results also support its potential unidimensional use for practical purposes, such as deriving a total HL score. Overall, the findings confirm that the HLS-EU-Q16 is a robust and adaptable instrument for assessing HL in Ecuadorian contexts and emphasize its relevance for public health strategies in Latin America.

Descriptive results revealed an overall mean HL score of 3.42 (SD = 0.54), suggesting a relatively adequate level in the sample. By dimensions, Health Promotion showed the highest mean (M = 3.54), while Disease Prevention obtained the lowest (M = 3.35). This pattern is consistent with previous studies indicating that preventive behaviors often face structural and sociocultural barriers limiting the understanding and application of preventive health information (11, 12). These findings highlight that HL is not only shaped by individual cognitive abilities, but also by the interaction between personal resources, healthcare system demands, and broader social contexts (7).

At the item level, participants reported greater ease in understanding health warnings related to risky behaviors (item 9, M = 3.64), suggesting that traditional messages discouraging harmful practices are more effectively received. Conversely, the lowest mean score was observed for item 8 (M = 3.10), which assessed the ability to find information on mental health issues such as stress or depression. This gap points to persistent challenges in accessing and comprehending mental health information, consistent with previous evidence showing limited HL in this area, particularly in contexts marked by stigma and insufficient specialized resources (13, 14).

The literature has consistently shown that higher HL levels are associated with better treatment adherence, improved quality of life, and appropriate use of healthcare services (4, 6, 9). However, HL is also strongly shaped by social determinants such as education, economic status, and type of health coverage (10, 14). In this sense, the scores observed in this study should be interpreted considering international findings that highlight how sociocultural, educational, and economic barriers hinder the comprehension and effective use of health information, beyond access to reliable sources (11, 12).

In Ecuador, these barriers are reflected in persistent gaps in access to curative services (32, 33), as well as inequalities driven by income, education, ethnicity, and geographic location (33, 34). Cultural and linguistic diversity, together with national language policies and structural limitations in rural areas, further deepen territorial inequalities (35, 39).

Taken together, these results reaffirm the need to design targeted interventions to strengthen HL in critical areas such as disease prevention and mental health, while addressing the social determinants and structural barriers that limit the full exercise of the right to health. They also highlight the importance of continuing to examine HL in diverse population subgroups, as recommended by recent regional studies (6, 13).

The confirmatory factor analysis demonstrated that all tested models achieved acceptable levels of fit, with the three-factor model (M3) showing the best performance. This model fully supported the original theoretical structure proposed by Sørensen et al. (1), yielding excellent goodness-of-fit indices (CMIN/DF = 2.366; CFI = 0.990; TLI = 0.988; RMSEA = 0.039; SRMR = 0.049), all within recommended thresholds. Furthermore, all factor loadings were positive and exceeded the 0.50 criterion, indicating strong associations between the items and their intended dimensions. These results provide solid evidence of convergent validity, confirming that each item reliably measures the construct it was designed to assess.

In light of the high intercorrelations among factors, these results should be interpreted as supporting both the multidimensional theoretical framework and the empirical possibility of a dominant general factor underlying HL. This interpretation aligns with previous studies that have observed comparable factor overlap while maintaining the conceptual three-domain structure for theoretical consistency.

These results are consistent with international validations in countries such as India, Bangladesh, and Romania, where the three-dimensional structure of the HLS-EU-Q16 has also been confirmed with high internal consistency (23, 25). The repeated identification of the three domains across diverse contexts strengthens the robustness of the theoretical framework underlying the instrument. Nonetheless, the literature has also reported alternative structures, such as two-factor models in Sweden (26) and unidimensional solutions in Brazil, Spain, and multicultural Swedish samples (21, 28, 29). Despite these variations, the present study aligns with the prevailing evidence supporting the multidimensional nature of health literacy, providing strong empirical support for the applicability of the HLS-EU-Q16 in the Ecuadorian context.

The present study provided robust evidence of strict factorial invariance of the HLS-EU-Q16 across sex, age groups, and area of residence, confirming that the instrument assesses the three dimensions of health literacy consistently in diverse sociodemographic subgroups. This ensures that observed differences reflect real variations in health literacy rather than measurement bias.

These findings are consistent with those reported in Venezuela by Francisco-Pérez et al. (41), where the scale also demonstrated invariance across sex, reinforcing its stability in Latin American contexts. By extending this evidence to age and residential area, the current study further supports the cross-group validity of the HLS-EU-Q16 in populations with heterogeneous demographic characteristics.

In contrast, evidence from France indicated that the scale was not fully invariant. Rouquette et al. (40) reported differential item functioning by sex, age, and education, showing that several items operated differently across groups. This suggests that comparisons in that context may be biased unless sensitivity analyses are performed. Together, the evidence highlights that while the HLS-EU-Q16 demonstrates strong measurement equivalence in Ecuador and Venezuela, cultural variability observed in France underscores the importance of local validation before generalizing results. The confirmation of strict invariance in this study provides methodological assurance, enabling valid comparisons across demographic groups and supporting the use of the scale in public health research and interventions in Latin America.

The HLS-EU-Q16 also showed high internal consistency in the Ecuadorian population, confirming the stability and accuracy of the overall scale and its three dimensions. These results align with the Venezuelan validation (41), though reliability indices were even higher in the present study, reinforcing the robustness of the instrument and its applicability across diverse sociocultural contexts in the region.

Construct validity was also confirmed, as the instrument differentiated health literacy levels across key sociodemographic groups in expected directions. Participants with postgraduate education consistently scored higher, reinforcing the well-documented association between education and the ability to access, understand, and use health-related information (1, 2, 6, 14, 17). Similar patterns have been observed in other Latin American studies, where education consistently emerges as a central determinant of HL (14, 18).

Disparities by region and area of residence further highlight how contextual factors shape HL. Individuals living in rural areas or in the Amazon region tended to present lower scores, reflecting persistent inequalities in access to health services and information in contexts with linguistic, cultural, and geographic barriers (11, 12, 16). These findings emphasize that HL extends beyond individual ability and is shaped by structural conditions.

Another relevant result concerns healthcare systems. Participants treated in private clinics had higher HL scores, suggesting that the quality of interactions with healthcare providers can either strengthen or hinder the development of health-related competencies (14). By contrast, individuals with chronic conditions scored lower, particularly in health promotion, highlighting persistent gaps in education and communication strategies for this population, despite evidence linking HL with better disease management and quality of life (4, 6, 9, 10).

Finally, financial hardship emerged as a critical determinant, with lower HL levels consistently reported by participants experiencing economic difficulties. This underscores how socioeconomic vulnerability not only restricts access to healthcare resources but also hinders the ability to process and apply health information effectively (11, 12, 14).

Taken together, these results provide robust support for the construct validity of the HLS-EU-Q16 and underscore the need for public health policies that address educational, regional, and socioeconomic disparities, promoting health literacy equitably across the Ecuadorian population.

### 4.1 Public health implications and future actions

Beyond its psychometric contribution, this study provides actionable insights for public health policy and practice. The lowest score in item 8, which measures the ability to find information on mental health issues (M = 3.10), reveals a critical gap in mental health literacy. This finding suggests the need for national and community-based initiatives to improve mental health literacy, reduce stigma, and enhance the accessibility and clarity of information related to psychological wellbeing (13, 14).

Similarly, lower HL scores associated with financial hardship, chronic illness, and rural residence highlight the importance of targeted and inclusive interventions. Public health strategies should prioritize these vulnerable groups by simplifying health information, ensuring linguistic and cultural appropriateness, and improving outreach through local health networks and digital platforms (11, 12, 33–35).

Furthermore, given the brevity, validity, and reliability of the HLS-EU-Q16, the Ministry of Health and academic institutions could integrate this tool into national health surveys and primary care programs to systematically identify individuals with limited HL. Such integration would facilitate the design of preventive and promotive health interventions and the monitoring of progress toward Sustainable Development Goals 2 (Zero Hunger) and 3 (Good Health and WellBeing).

In summary, the validated HLS-EU-Q16 not only serves as a reliable assessment tool but also as an evidence-based instrument to guide equity-oriented public health policies, enhance community empowerment, and promote informed decision-making in health contexts.

### 4.2 Limitations

This study has several limitations that should be acknowledged. The use of a non-probabilistic convenience sample constitutes the main methodological limitation, as the sample was predominantly female, urban, young, and highly educated, which does not mirror the demographic structure of the Ecuadorian population. Specifically, the sample showed an overrepresentation of women (64.22%), a relatively young mean age (30.97 years) with limited participation of older adults, a high educational level (65.69% with technical or higher education), a predominance of individuals self-identifying as Mestizo (86.76%), and lower representation of ethnic minorities. From a geographic perspective, participants from the Highlands (55.72%) and the Amazon region (36.93%) were overrepresented, whereas the Coast (7.35%) was underrepresented; additionally, most respondents resided in urban areas (77.12%). While this composition allows for meaningful analysis of health literacy across several Ecuadorian subgroups, the results should be interpreted as primarily reflecting a young, urban, more educated, and Mestizo population. Therefore, caution is warranted when generalizing the findings to men, older adults, coastal populations, individuals with lower educational attainment, and ethnic minorities.

In addition, participation was voluntary and recruitment relied largely on online dissemination strategies, although these were complemented by in-person recruitment in community settings. Under these conditions, self-selection bias cannot be excluded, as individuals with greater interest in health-related topics, higher digital literacy, or greater health awareness may have been more inclined to participate. As a result, estimates of mean health literacy levels should be interpreted prudently.

The cross-sectional nature of the study also restricts the assessment of test–retest reliability and predictive validity. Longitudinal research designs are required to examine the temporal stability of the HLS-EU-Q16 and its ability to predict relevant health outcomes, such as adherence to treatment or engagement in preventive behaviors.

Furthermore, construct validity was primarily examined through known-groups comparisons, without incorporating convergent or discriminant validity evidence. Future studies should address this limitation by correlating the HLS-EU-Q16 with functional health literacy measures (e.g., the Short Test of Functional Health Literacy in Adults [S-TOFHLA] or the Newest Vital Sign) as well as with theoretically distinct constructs, such as personality traits, to provide a more comprehensive validation framework.

Finally, as with all self-report instruments, potential response biases including social desirability and recall bias cannot be ruled out. Subsequent research would benefit from incorporating qualitative approaches, as well as measures of usability and acceptability, to complement the quantitative psychometric evidence and deepen understanding of individuals' health literacy experiences.

## 5 Conclusion

This study confirms that the Spanish version of the HLS-EU-Q16 is a valid and reliable instrument for assessing health literacy in Ecuador. The results provide strong evidence supporting its three-factor structure and internal consistency, aligned with the original theoretical model. Importantly, this research represents the first formal validation of a health literacy tool in the country, rather than a population-level assessment of HL. Therefore, the findings should be interpreted with caution, as the non-probabilistic convenience sample was not representative of the Ecuadorian adult population. Future research using probabilistic and nationally representative samples is needed to accurately estimate HL levels and examine regional, socioeconomic, and educational disparities across Ecuador.

The validated HLS-EU-Q16 can serve as a foundational tool for national and regional surveillance of health literacy. Its integration into health monitoring systems would support evidence-based policy design, helping to reduce social and regional inequalities in access to health information and services. Furthermore, the availability of a culturally adapted and psychometrically robust instrument enables policymakers and public health professionals to identify vulnerable groups, monitor the impact of health education programs, and promote actions aimed at strengthening preventive and promotive health capacities across populations.

A key strength of the HLS-EU-Q16 is that it is a brief and easy-to-administer scale, which makes it highly practical for both research and applied settings. Its simplicity facilitates implementation in population-based surveys, clinical contexts, and community programs without imposing excessive demands on respondents or practitioners.

Beyond its methodological contribution, the validated HLS-EU-Q16 provides a strategic foundation for advancing health equity in Latin America. By enabling cross-country comparisons and standardized measurement, it supports regional collaboration in health literacy research and evidence-based policy development. The instrument can guide the design, implementation, and monitoring of strategies aimed at strengthening disease prevention, promoting health, and reducing inequalities in access to health information. Moreover, its validation in Ecuador provides a foundation for future studies that can expand representativeness and contribute to building robust national data on health literacy. Its use in Ecuador also contributes to regional comparability of health literacy research in Latin America, supporting the development of more inclusive and equitable health systems.

## Data Availability

The raw data supporting the conclusions of this article will be made available by the authors, without undue reservation.
