# Supplementary material for: Psychometric validation of the Spanish HLS-EU-Q16 in Ecuador: evidence for health literacy assessment and public health education
Source: Front Public Health. 2026 Feb 11;14:1717946. doi: 10.3389/fpubh.2026.1717946 (PMC12932619; doi:10.3389/fpubh.2026.1717946)
Supplement: Supplementary file 1 [file Data_Sheet_1.pdf]

ESCALA DE ALFABETIZACIÓN EN SALUD APLICADA EN LA POBLACIÓN ECUATORIANA/ HEALTH LITERACY SCALE APPLIED TO THE ECUADORIAN POPULATION

Para las siguientes preguntas, por favor elija una de las opciones que describa el grado de facilidad o dificultad que usted experimenta en determinadas actividades relacionadas con la salud.

Actividades	Muy dificil	Difícil	Ni fácil ni difícil	Fácil	Muy fácil
1. Encontrar información sobre las enfermedades que le interesa conocer.					
2. Averiguar dónde obtener ayuda profesional cuando está enfermo/a (p. ej., médico, farmacéutico, psicólogo).					
3. Entender lo que dice el médico.					
4. Entender las indicaciones del médico o del farmacéutico sobre cómo tomar los medicamentos prescritos.					
5. Identificar cuándo necesita una segunda opinión médica sobre asuntos relacionados con su salud.					
6. Utilizar la información que le brinda su médico para ayudarle a tomar decisiones sobre su enfermedad.					
7. Seguir las indicaciones de su médico o farmacéutico.					
8. Encontrar información sobre cómo afrontar problemas de salud mental como el estrés o la depresión.					
9. Entender las advertencias de salud relacionadas con hábitos como fumar, hacer poca actividad física o consumir alcohol en exceso.					
10. Entender por qué necesita realizarse detección temprana de enfermedades o chequeos médicos (p. ej., mamografía, prueba de glucosa y prueba de presión arterial).					
11. Identificar si la información que aparece en los medios es confiable (p. ej., televisión, internet u otros medios de información).					
12. Decidir cómo protegerse de enfermedades con base en la información proporcionada por los medios (p. ej., periódicos, folletos, internet y otros medios).					
13. Encontrar actividades que sean buenas para su bienestar mental (p. ej., meditación, ejercicio, caminar, pilates, etc.).					
14. Entender los consejos de salud brindados por familiares y amigos.					
15. Entender la información proporcionada por los medios sobre cómo mejorar su salud.					
16. Identificar cuáles de sus hábitos diarios afectan su salud (p. ej., consumo de alcohol, hábitos alimentarios, ejercicio, etc.).					

For the following questions, please choose one of the options below that describe the degree of ease or difficulty you experience in certain health-related activities.

Actividades	Very Difficult	Difficult	Neither too easy nor too difficult	Easy	Very easy
1. Find information about the diseases you are interested in learning about.					
2. Find out where to get professional help when you are sick (e.g., doctor, pharmacist, psychologist).					
3. Understanding what the doctor says					
4. Understand the doctor's or pharmacist's instructions on how to take prescribed medications.					
5. Identify when you need another doctor's opinion on health-related issues					
6. Use the information your doctor gives you to help you make decisions about your illness.					
7. Follow your doctor's or pharmacist's instructions					
8. Find information on how to deal with mental health problems such as stress or depression.					
9. Understand health warnings related to habits such as smoking, low physical exercise or excessive alcohol consumption.					
10. Understand why you need to have early disease screening or medical check-ups (e.g., mammogram, blood sugar and blood pressure test).					
11. Identify whether the information appearing in the media is reliable (e.g., television, Internet, or other information media).					
12. Decide how to protect themselves from disease based on information provided by the media (e.g., newspapers, brochures, Internet and other information media).					
13. Find activities that are good for your mental well-being (e.g., meditation, exercise, walking, pilates, etc.).					
14. Understand the health advice given by family and friends.					
15. Understand information provided by the media on how to improve their health					
16. Identify which of your daily habits affect your health (e.g., habits related to alcohol consumption, eating habits, exercise, etc.).					
